# Effects of caloric restriction on the gut microbiome are linked with immune senescence

**DOI:** 10.1186/s40168-022-01249-4

**Published:** 2022-04-04

**Authors:** Julia Sbierski-Kind, Sophia Grenkowitz, Stephan Schlickeiser, Arvid Sandforth, Marie Friedrich, Désirée Kunkel, Rainer Glauben, Sebastian Brachs, Knut Mai, Andrea Thürmer, Aleksandar Radonić, Oliver Drechsel, Peter J. Turnbaugh, Jordan E. Bisanz, Hans-Dieter Volk, Joachim Spranger, Reiner Jumpertz von Schwartzenberg

**Affiliations:** 1 Department of Endocrinology and Metabolism, Charité-Universitätsmedizin Berlin, Corporate Member of Freie Universität Berlin, Humboldt-Universität zu Berlin, Chariteplatz 1, 10117 Berlin, Germany; 2 Berlin Institute of Health (BIH), Berlin, Germany; 3 BIH Center for Regenerative Therapies (BCRT), Charité - Universitätsmedizin Berlin and Berlin Institute of Health (BIH), Berlin, Germany; 4 Institute for Diabetes Research and Metabolic Diseases of the Helmholtz Center Munich at the University of Tübingen, Tübingen, Germany; 5 Berlin Institute of Health at Charité - Universitätsmedizin Berlin, Flow & Mass Cytometry Core Facility, Berlin, Germany; 6 Medical Department for Gastroenterology, Infectious Diseases and Rheumatology, corporate member of Freie Universität Berlin, Humboldt-Universität zu Berlin, Berlin, Germany; 7 DZHK (German Centre for Cardiovascular Research), partner site Berlin, Berlin, Germany; 8 Robert Koch Institute, Berlin, Germany; 9 Department of Microbiology & Immunology, University of California San Francisco, San Francisco, CA USA; 10 Department for Biochemistry and Molecular Biology, Pennsylvania State University, University Park, PA USA; 11 Institute of Medical Immunology, Charité - Universitätsmedizin Berlin, corporate member of Freie Universität Berlin, Humboldt-Universität zu Berlin, Berlin, Germany; 12 Department of Internal Medicine IV, Division of Diabetology, Endocrinology and Nephrology, Eberhard-Karls University of Tübingen, Tübingen, Germany; 13 Cluster of Excellence EXC 2124 Controlling Microbes to Fight Infections, University of Tübingen, Tübingen, Germany; 14 German Center for Diabetes Research (DZD e.V.), Neuherberg, Germany

**Keywords:** Gut microbiota, Adaptive immune system, Caloric restriction, Obesity, Immune senescence

## Abstract

**Background:**

Caloric restriction can delay the development of metabolic diseases ranging from insulin resistance to type 2 diabetes and is linked to both changes in the composition and metabolic function of the gut microbiota and immunological consequences. However, the interaction between dietary intake, the microbiome, and the immune system remains poorly described.

**Results:**

We transplanted the gut microbiota from an obese female before (AdLib) and after (CalRes) an 8-week very-low-calorie diet (800 kcal/day) into germ-free mice. We used 16S rRNA sequencing to evaluate taxa with differential abundance between the AdLib- and CalRes-microbiota recipients and single-cell multidimensional mass cytometry to define immune signatures in murine colon, liver, and spleen. Recipients of the CalRes sample exhibited overall higher alpha diversity and restructuring of the gut microbiota with decreased abundance of several microbial taxa (e.g., *Clostridium ramosum*, *Hungatella hathewayi*, *Alistipi obesi*)*.* Transplantation of CalRes-microbiota into mice decreased their body fat accumulation and improved glucose tolerance compared to AdLib-microbiota recipients. Finally, the CalRes-associated microbiota reduced the levels of intestinal effector memory CD8^+^ T cells, intestinal memory B cells, and hepatic effector memory CD4^+^ and CD8^+^ T cells.

**Conclusion:**

Caloric restriction shapes the gut microbiome which can improve metabolic health and may induce a shift towards the naïve T and B cell compartment and, thus, delay immune senescence. Understanding the role of the gut microbiome as mediator of beneficial effects of low calorie diets on inflammation and metabolism may enhance the development of new therapeutic treatment options for metabolic diseases.

**Trial registration:**

NCT01105143, “Effects of negative energy balance on muscle mass regulation,” registered 16 April 2010.

**Video Abstract**

**Supplementary Information:**

The online version contains supplementary material available at 10.1186/s40168-022-01249-4.

## Background

The incidence of obesity is continuously increasing, affecting more than 2 billion people worldwide [1]. Obesity is associated with cardiovascular diseases, such as hypertension, peripheral artery disease, myocardial infarction, and metabolic comorbidities, ranging from insulin resistance to dyslipidemia, non-alcoholic steatohepatitis, and type 2 diabetes [2–5]. Obesity has been widely associated with systemic and adipose tissue inflammation with accumulating pro-inflammatory T cells, including cytotoxic CD8^+^ T cells, CD4^+^ type 1 helper T cells (Th1), CD4^+^ type 3/17 helper T cells (Th17), and memory B cells that exacerbate inflammation through the recruitment of chemokines, initiating an inflammatory process that promotes insulin resistance [6–10]. Moreover, obesity-associated chronic low-grade inflammation has been shown to impair insulin sensitivity through activation of c-Jun N-terminal kinase and nuclear factor-kappa B signaling pathways that subsequently increase the release of proinflammatory cytokines such as tumor necrosis factor-alpha and interleukin-6 (IL-6) [9]. We and others previously reported that the T cell profile in visceral and subcutaneous adipose tissue is associated with insulin resistance and systemic inflammation in humans and that insulin resistance correlates with a shift towards the memory T cell compartment in adipose tissues [6, 11–13]. The relative increase in the frequency of memory cells is a process, referred to as immune senescence, an age-associated immune alteration, which can be delayed by caloric restriction [14, 15]. Importantly, the trillions of microbes colonizing the gastrointestinal tract, the gut microbiota, can also modulate adipose tissue expansion and glucose metabolism, which has been shown in various colonization experiments in germ-free (GF) mice [16, 17]. Numerous clinical studies revealed an altered microbiota composition in obese participants with type 2 diabetes, and, thus, emphasized a critical role of the gut microbiota on metabolic homeostasis [18–20]. Obesity is linked to profound alterations in gut microbial communities in humans and mice. However, caloric restriction-induced weight loss can reverse this phenotype [17, 21, 22]. We have recently reported that the gut microbiota shows highly dynamic responses to dietary changes and is causally linked to nutrient absorption in humans [23]. Diet is one of the strongest drivers for changes in the microbial community structure which even dominates the genetic background of the host [24]. Colonization of GF mice with a microbiota harvested from genetically obese *ob/ob* mice resulted in higher percentage of total body fat compared to microbiota recipients from lean donors [17], emphasizing the critical role of the gut microbiota for energy balance regulation. Experiments in GF and gnotobiotic mice have shown that the host immune system is distinctly shaped by the gut microbiota [25]. For example, it has been reported, that the microbial composition regulates the balance of Th17 and regulatory T cells in the lamina propria of the small intestine [26]. Recently, another group identified the immunomodulatory effects of diverse gut microbes in mice monocolonized with 53 individual bacterial species, systemically analyzing host immunologic adaptation to colonization [27]. However, the interaction of the gut microbiota with innate and adaptive immune cells remains unclear with limited data and controversial results being reported. Altogether, the impact of diet-induced obesity, dietary interventions, as well as caloric interventions on the gut microbiome, is well described [16, 17, 28–32], but downstream consequences of diet-driven microbiome alterations on host immune signatures, are yet unclear and remain to be elucidated.

In this study, we combined a human dietary intervention trial with gnotobiotic experiments applying immunophenotyping using single-cell multidimensional mass cytometry with a 31-antibody panel consisting of leukocyte subset differentiation markers. Together, our results emphasize the importance of the gut microbiota for mediating the host response to dietary interventions.

## Methods

### Clinical study protocol

Fecal samples for fecal microbiota transfer (FMT) were collected from one of the top 5 weight losers during a weight loss intervention study focusing on muscle mass regulation in postmenopausal women “Effects of negative energy balance on muscle mass regulation” (registered at https://clinicaltrials.gov, NCT01105143) at the Department of Endocrinology of the Charité- Universitätsmedizin, Berlin, Germany [33, 34].

This study was carried out in accordance with the recommendations of the International Conference on Harmonization Guidelines for Good Clinical Practice and the Declaration of Helsinki. The protocol of the study was approved by the local Ethics Committee of the Charité- Universitätsmedizin Berlin (EA2/050/10). All subjects gave written informed consent before participating in this study. Inclusion criteria comprised female gender, a BMI > 27 kg/m^2^, and postmenopausal status. Severe untreated medical, neurological, and psychiatric diseases within the last 5 years which may interfere with the planned interventions, such as unstable coronary heart disease, kidney and liver disease, systemic infections, endocrinological disorders, and hypertension (systolic blood pressure > 180 mm Hg, diastolic blood pressure > 110 mm Hg) were excluded by medical history assessment. Further exclusion criteria were changing dieting or smoking habits significantly in the last 2 months including a weight loss of 5 kg or more. Exclusion criteria for participants were also a history of medication, changes in smoking habits, or diets within the last 3 months, which may have significantly affected body weight. Participants with synthetic thyroid medications were not excluded if they were clinically euthyroid. A total of 80 overweight or obese female subjects were initially included in the study. Subjects were randomly assigned to the intervention and the control group, respectively.

The detailed study protocol has been reported elsewhere [11, 34, 35]. In brief, weight loss was induced by an established, standardized weight reduction program for 12 weeks in the intervention group. In the first 8 weeks of the 12-week calorie restriction period, weight loss was performed by a very-low-calorie diet (VLCD, 800 kcal/d) replacing all meals with a formula diet (Optifast 2®, Nestlé HealthCare Nutrition GmbH) [36]. Before and after these eight weeks of VLCD, stool samples were taken for downstream analyses and experiments from the one individual of the top 5 weight losers, who exhibited the strongest improvement in insulin sensitivity.

### Mice and intervention protocol

Male GF C57BL/6J mice (15–24 weeks old, *n* = 33) were housed in gnotobiotic isolators with two to four mice per cage on a 12-h light-dark cycle. Mice were divided into three groups, each group in one individual isolator, respectively. Mice were colonized by FMT with a human gut microbiota, that was collected before (AdLib, *n* = 13) and after (CalRes, *n* = 9) a calorie-restricted dietary intervention and compared to an age-matched GF control group (*n* = 11). Animals were maintained on a chow diet (SSNIFF, V1534-300) which provides 9% kJ from fat, 33% kJ from proteins and 58% kJ from carbohydrates for 3 weeks after the FMT. Mice were sacrificed by cervical dislocation following anesthesia. This study was carried out in accordance with the Guide for the Care and Use of Laboratory Animals of the National Institutes of Health and the Animal Welfare Act under the supervision of our institutional Animal Care and Use Committee. Animal protocols were conducted according to institutional ethical guidelines of the Charité-Universitätsmedizin, Berlin, Germany, and were approved by the Landesamt für Gesundheit und Soziales (approval number G 0085/17 LAGeSo Berlin, Germany) and comply with the ARRIVE guidelines.

### Human FMT into germ-free mice

Human stool samples were collected before starting the intervention (AdLib) and after 8 weeks on the formula diet in the intervention group (CalRes). As previously described [34], volunteers were given a stool collection kit and were instructed to store collected stool samples in the freezer at − 20 °C until they were transported in a cooled container to our research unit. All samples were subsequently stored at − 80 °C. Each fecal sample was thawed and prepared in an anaerobic chamber. Stool samples were resuspended in phosphate-buffered saline (PBS) (1:10, Sigma-Aldrich), followed by centrifugation for 1 min at 500 rpm. The resulting preparation was externally sterilized, transferred into gnotobiotic isolators, and 200 μl were administered by oral gavage. Mice not receiving the FMT received oral gavages of autoclaved drinking water as a sham gavage. For the FMT, samples were chosen from an individual who was the top five weight losers of the program, who showed the strongest increase in insulin sensitivity during the weight loss phase and provided a complete set of stool samples (since not all individuals provided stool samples at each study visit).

### Analysis of metabolic parameters

Mice were weighed every two days throughout the course of the experiment using a model *EMB 200-*2 scale (KERN & Sohn GmbH)*.* Mice were fasted for 6 hours, and an oral gavage glucose tolerance test (OGTT) was performed. Fasted blood glucose levels were determined before a solution of glucose (10% Glucose, Braun) was administered (2 g glucose/kg body weight) by oral gavage. Subsequently, blood glucose levels were monitored at 15, 30, 60, and 120 min after glucose administration. Total fecal excretion and food consumption were obtained weekly from one representative cage per group. The energy density of the chow diet and fecal samples were determined using bomb calorimetry.

### Bomb calorimetry

The energy content of the chow diet and mouse feces were analyzed using an Isoperibol Calorimeter 6200 instrument with a model 1108 oxygen bomb (Parr Instrument Co.), as described elsewhere. Briefly, the sample was pressed into a 1-g pellet and was placed into the bomb, which was filled with oxygen (3000 kPa), and placed in a bomb cylinder with 2000 ml distilled water. The increase in the temperature (∆*T*) of the surrounding water by the heat produced at combustion was measured. The energy content (*E*) of the pellet was calculated as follows:

$$E=W\times \Delta T/m$$

The energy equivalent (W) specifies the energy required to raise the temperature of the surrounding water by 1 °C (*W* = [Calorie/°C]).

### Fecal DNA extraction and sequencing

Fecal samples were collected from mice in the AdLib and the CalRes groups respectively at day 1, 3, 7, 14, and 21 after colonization with the human donor microbiota. DNA was extracted using the QIAamp Fast DNA Stool Mini Kit as detailed in the manufacturer’s protocol (Qiagen, USA). Library preparation for 16S rRNA gene sequencing was done according to the protocol of Illumina (USA), targeting the 16S V3 and V4 region, and sequenced on an Illumina MiSeq instrument with 2 × 300-bp v3 chemistry. Reads were demultiplexed using bcl2fastq (Illumina, San Diego, USA) and submitted to initial quality control by QCumber-2 (https://gitlab.com/RKIBioinformaticsPipelines/QCumber). In brief this pipeline trims sequencing adapters as well as low quality read ends and discards reads shorter than 50bp using Trimmomatic [37].

### 16S rRNA sequencing analyses

Demultiplexed reads were processed and denoised by *DADA2* [38]. Taxonomy was assigned using the *DADA2* implementation of the RDP classifier [39] using the *DADA2*-formatted training sets for SILVA138 (zenodo.org/record/3731176/files/silva_nr_v138_train_set.fa.gz). Species were assigned by exact matching against a reference (zenodo.org/record/3731176/files/silva_species_assignment_v138.fa.gz). A phylogenetic tree was constructed de novo via the *DECIPHER* and *phangorn* R packages. The optimized tree counts for a total of 1504 detected different amplicon sequence variants (ASVs), and taxonomy tables were converted into a *phyloseq* object [40] for further downstream analyses. No filtering except for unassigned taxa was performed prior to calculation of diversity metrics and ordination analysis of the complete dataset (1414 ASVs, 104 samples, day 1 to 21), while singletons were removed from differential abundance analysis in 20 samples at day 21 (284 ASVs).

### Isolation of murine splenic immune cells

Spleen was homogenized, passed through 70 μm filters, washed, and subjected to red blood cell lysis (ACK lysing buffer, GIBCO). The red blood cell lysis was stopped by adding washing buffer (MaxPar Cell staining Buffer, Fluidigm), and the homogenate was then passed through 30 μm filters and washed again before final suspension in MaxPar Cell staining Buffer. The cell suspension was adjusted to 2x10^6^ cells/500 μl.

### Isolation of murine intrahepatic immune cells

Isolation of murine intrahepatic immune cells was performed as reported previously [41]. Briefly, whole liver was prepared by harvesting perfused liver lobes into 15 ml PBS. The liver was dissociated mechanically, followed by tissue digestion (0.5 mg/ml Collagenase Type IV (Worthington), 0.02 mg/ml DNAse I (Sigma-Aldrich), 2 % fetal calf serum, 0.6 % bovine serum albumin in HBSS) for 30 min at 37 °C in a rotation shaker (200 rpm). Hepatocytes were then pelleted (30 g, 1 min, room temperature (RT)), the supernatant was centrifuged (310 g, 4 min, 4 °C), and the pellet was resuspended in 30 % density gradient media (Percoll, Sigma-Aldrich) in HBSS followed by centrifugation (800 g, 30 min, RT) to enrich liver mononuclear cells. Following red blood cell lysis (ACK lysing buffer, GIBCO), the homogenate was washed again, and passed through 30-μm filters before final suspension in MaxPar Cell staining Buffer. The cell suspension was adjusted to 2 × 10^6^ cells/500 μl.

### Isolation of lamina propria mononuclear cells

Lamina propria mononuclear cells (LPMC) were isolated from colon sections (10 cm distal part) as described previously [42]. Briefly, the colon was opened longitudinally, cut into small pieces, and subsequently incubated twice with HBSS containing 1 mmol/l EDTA for 30 min, followed by enzymatic digestion (0.44 mg/ml Collagenase D and 20 μg/ml DNase I in RPMI, Sigma-Aldrich) for 60 min at 37 °C. After a filtration step through a 100μm filter, the LPMC were purified by 44/67% density gradient (Percoll™, GE Healthcare) centrifugation for 20 min at 600 × g.

### Staining for mass cytometry

For barcoding, anti-CD45 antibodies were conjugated in house to 89Y, 147Sm, and 166Er. Up to six individual samples were stained with a combination of the different anti-CD45 antibodies for 30 min at 4 °C. Cells were then washed and pooled for surface staining. A total of 2 × 10^6^ cells per sample were stained in a 96-deep-well plate with metal-conjugated antibodies (antibodies for mass cytometry as previously described [43]) for 30 min at RT. 0.5 mM cisplatin (Fluidigm) was added as live/dead cell marker for the last 10 min. Cells were then washed twice and the pellet was resuspended in 1 ml of nucleic acid Intercalator-Ir solution (12.5 nM Cell-ID Intercalator-Ir diluted in MaxPar Fix and Perm Buffer, Fluidigm), followed by incubation at RT for 30 min. Followed by two washing steps, the cells were resuspended in 100 μl formaldehyde-solution (1:10) and fixed overnight at 4 °C. The next day, cells were washed twice with ultrapure water and kept at 4 °C until mass cytometry measurement.

### Mass cytometry measurement

Cells were analyzed using a CyTOF2 upgraded to Helios specifications, with software version 6.7.1014, using a narrow bore injector. The instrument was tuned according to the manufacturer’s instructions with tuning solution (Fluidigm) and measurement of EQ four element calibration beads (Fluidigm) containing ^140/142^Ce, ^151/153^Eu, ^165^Ho, and ^175/176^Lu served as a quality control for sensitivity and recovery. Directly prior to analysis, cells were resuspended in ddH_2_O, filtered through a 20-μm cell strainer (Celltrics, Sysmex), counted and adjusted to max. 8 × 10^5^ cells/ml. EQ four element calibration beads were added at a final concentration of 1:10 v/v of the sample volume to be able to normalize the data to compensate for signal drift and day-to-day changes in instrument sensitivity. Samples were acquired with a flow rate of max. 300 events/s. The lower convolution threshold was set to 400, with noise reduction mode turned on and cell definition parameters set at event duration of 10-150 pushes (push = 13 μs). The resulting flow cytometry standard (FCS) files were normalized and randomized using the CyTOF software’s internal FCS-Processing module on the non-randomized (“original”) data. The default settings in the software were used with time interval normalization (100 s/minimum of 50 beads) and passport version 2. Intervals with less than 50 beads per 100 s were excluded from the resulting FCS file.

### Mass cytometry data analysis

FCS files were compensated for signal spillover using *CATALYST* package and per channel intensity ranges were aligned between batches of measurements using the normalizeBatch function (*cydar* package). Cytobank [44] was used for manual debarcoding, gating of lymphocyte subsets and to perform viSNE on pre-gated subsets as described previously [43]. For each organ (colon, liver, spleen) semi-supervised population identification was conducted by graph-based clustering (*R phenograph* package) on total leukocytes as well as on events pre-gated for TCRab^−^CD19^−^NK1.1^−^ and TCRab^+^ to better resolve the innate/myeloid compartment and T cells, respectively. Leaf nodes were merged into biologically relevant subsets by second-level hierarchical clustering while putting more weight on lineage-delineating markers.

### Statistical analysis

The results are shown as the mean ± SD. A *P*-value of < 0.05 was considered significant. All analyses were performed using GraphPad Prism version 7 (GraphPad Software) and R version 3.4.0, available free online at https://www.r-project.org. Mathematical correction for multiple comparisons was made whenever indicated. Diversity metrics were calculated from microbial ASVs and principal coordinate analysis (PCoA) carried out using the *phyloseq* and *vegan* packages. Changes in alpha-diversity over time and between group were tested by ANOVA-type statistic using the *nparLD* package [45]. Bray-Curtis dissimilarity computed from variance-stabilized counts of the complete dataset was used to quantify and visualize compositional changes between microbial communities by PCoA. Global differences between groups and changes over time were tested on the dissimilarity matrix by permutational analysis of variance using *adonis* with 9999 replications. Differential abundance analysis of ASVs between CalRes and AdLib groups at day 21 was carried out using DESeq2. Abundances of significant ASVs (FDR < 0.01) were visualized by heatmap along with log2-fold change (LFC) values.

For statistical analysis of cell population abundances, we fitted a generalized linear mixed-effects model (GLMM) for each population using the lme4 package as previously described [46]. To take into account the day-to-day (batch) variability of the mass cytometry runs, we included batch as fixed effect in the models and all quantitative data presented are shown after batch-adjustment. *P*-values resulting from differential abundance testing were adjusted using the Benjamini-Hochberg procedure and an FDR-cutoff of 5% across all clusters/subsets and between-group comparisons. To estimate differences between manually gated immune cell subsets, one-way ANOVA followed by Tukey’s tests were applied.

To investigate associations between microbial composition and immune cell populations, we performed sparse canonical correlation analysis. For each organ, a semiparametric correlation matrix was estimated based on the latent Gaussian copula model using the *mixedCCA* package with selection of canonical correlation vectors using L1-penalization (*lasso*) and the Bayesian information criterion for unknown error variance [47]. Sparse canonical covariates were computed by matrix multiplication of the ranked variables of each dataset with its canonical vector. Each latent correlation matrix was ordered using the projection on its first principal component to visualize the cross-correlation structures and to additionally highlight the top ten taxa that either positively or negatively associate with the immunological datasets. All heatmaps and circular correlation plots were generated using the *ComplexHeatmap* and *circlize*.

The body weight and glucose time course data were analyzed by two-way repeated measures ANOVA followed by post-hoc (Bonferroni) test.

## Results

### Caloric restriction changes the gut microbiome composition in humanized gnotobiotic mice

**Fig. 1:**
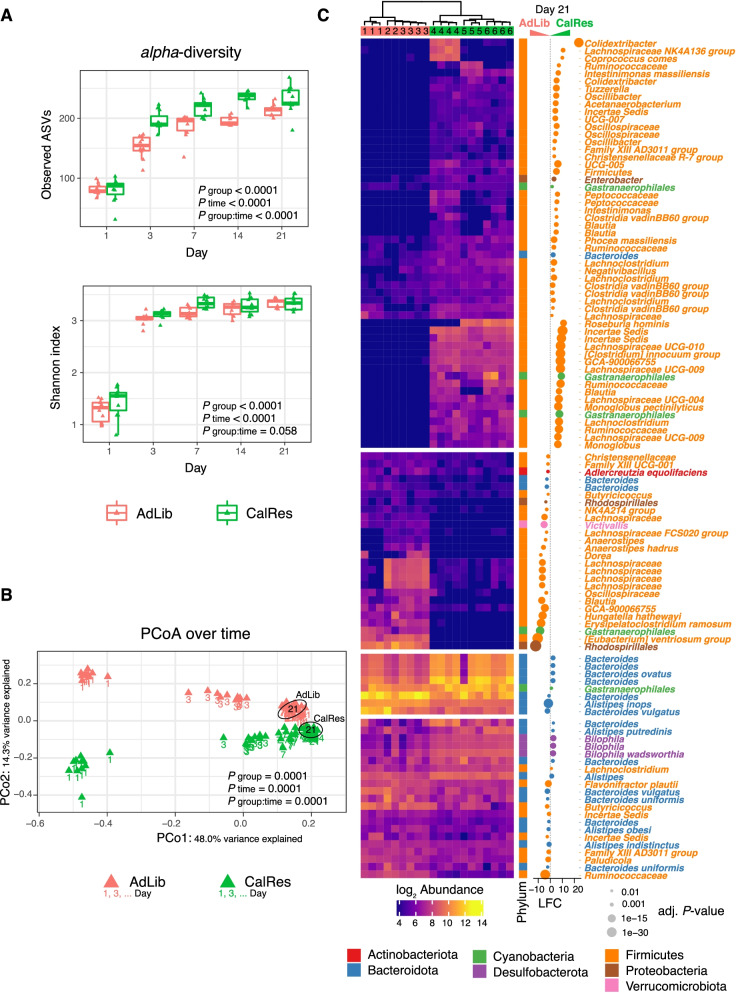
Caloric restriction changes the gut microbiome composition in humanized gnotobiotic mice. **A** Number of observed amplicon sequence variants (ASVs) and Shannon index indicate microbial richness and diversity for 3 weeks following colonization. *P*-values are given for repeated measures non-parametric ANOVA-type testing for differences between groups and time points (*n* = 104 stool samples across 23 mice and 5 time points). **B** Principal coordinates of Bray-Curtis dissimilarity between stool samples. Color indicates groups. Numeric labels denote the 5 time points. Ellipses enclose group masses (99% CI) for day 21. *P*-values are given for *adonis* permutational analysis of variance testing for group × time differences. **C** Heatmap representation of day 21 differentially abundant ASVs (*n* = 20). ASVs are shown at the most specific assigned taxonomy with FDR-adjusted *P*-values < 0.01 in DESeq2 two-sided Wald test. Bubbles indicate log-fold change (LFC) between the two groups. Heatmap colors indicate relative abundances as variance-stabilizing (log2) transformed counts ranging from 4 (dark blue, corresponding to 0 raw counts) to 14 (yellow, corresponding to 14K raw counts). Color of bubbles and species labels denote phylum. Bubble sizes are proportional to − log10 FDR-adjusted *P*-values. Dendrogram leaf numbers indicate individual housing cages

Germ-free mice were colonized with the human gut microbiota taken from an obese female before (AdLib) and after (CalRes) an 8-week very low-calorie diet (800 kcal/day), which was part of a randomized human intervention study, including 80 postmenopausal women who were overweight or obese [11, 33, 34]. In our prior work, we have shown that caloric restriction has pronounced impacts on microbial community structure with marked changes in metabolic activity and expansion of bacteria that can degrade host glycans [34]. Importantly, differences between the microbiota of humans before and after caloric restriction were recapitulated in microbiota from gnotobiotic-recipient mice [34]. Here, we collected stool samples from GF-recipient mice frequently over a period of 3 weeks and performed 16S rRNA gene sequencing to determine differences in microbiota between groups. Consistent with previous findings [48], the CalRes-recipient microbiota exhibited overall higher alpha diversity compared to the AdLib group (Fig. 1A). A principal component analysis revealed that the first two components driving the variation within the microbiota are time after colonization and donor group (Fig. 1B), demonstrating the development of different microbial communities between groups (ADONIS *p*_group:time_ < 0.001). Additionally, these data demonstrate that one week is sufficient for a human microbiota to mature in a previously GF mouse gut, replicating, as shown previously [34, 49]. Using DESeq2 differential abundance testing, AdLib and CalRes samples were distinguished by defined microbial taxa as shown in Fig. 1C. Colonization with the CalRes microbiota led to a significant decrease of multiple taxa such as *Rhodospirillales*, *Eubacterium ventriosum*, *Hungatella hathewayi*, or *Chlostridium ramosum* [50–54]. *Rhodospirillales* belong to the phylum Proteobacteria, which was overrepresented in obese humans in multiple studies [55, 56], whereas several bacteria that belong to the Lachnospiraceae family and were shown to negatively correlate with energy consumption [57] were significantly more abundant in the CalRes group. Interestingly, the abundance of *Monoglobus pectinilyticus*, which possesses a highly specialized glycobiome for pectin degradation was significantly increased in mice that had been inoculated with the CalRes microbiota. *M. pectinilyticus* is present in humans with a high intake of dietary fiber and pectin [58]. Recipients of the CalRes sample showed increased abundance of several taxa belonging to the phylum Bacteroidetes in line with previous findings [17, 31], whereas other bacteria specialized for the metabolism of plant polysaccharides (*Ruminococcus*, *Eubacterium*) [59, 60] were decreased, consistent with our findings in the human microbiota [34]. A comprehensive list of differentiating taxa is given in Additional file 2. Together, these results suggest that inoculation of the AdLib or the CalRes human microbiota into GF mice induced different shifts in recipient gut microbial communities. Importantly, given the limitation of a single donor approach, comparison of our selected donor (MMSP50) against the remainder of the cohort indicated that the donor was a good representative of the cohort [34] (Supplementary Figure 1A-C).

### Colonization with CalRes-associated gut microbiota alters body fat and glucose clearance

**Fig. 2:**
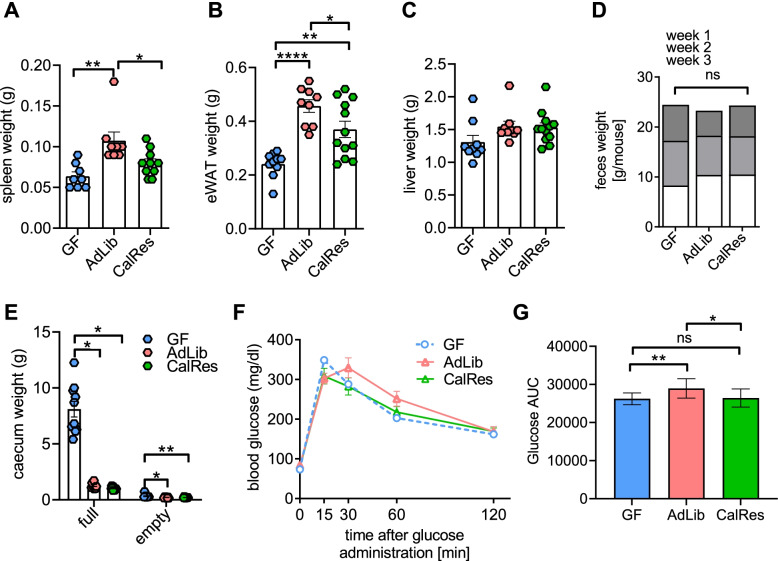
Colonization with CalRes-associated gut microbiota alters body fat and glucose clearance. Metabolic analysis of germ-free (GF) mice and mice inoculated with AdLib and CalRes human gut microbiota. **A–C** Spleen (**A**), epigonadal white adipose tissue (eWAT) (**B**), and liver (**C**) weights from GF and colonized mice. **D** Feces weight was measured using bomb calorimetry in GF and colonized mice. **E** Caecum weights from GF and colonized mice were analyzed with and without fecal contents. **F** Fasting male adult GF or colonized mice maintained on normal chow diet were challenged with oral glucose and blood was sampled for glucose at times indicated. **G** Area under the OGTT glucose-time curve (AUC). * *P* < 0.05, ** *P* < 0.01, **** *P* < 0.0001 as determined using ANOVA with Bonferonni’s post-test correction for multiple comparisons; error bars = SEM; ns = not significant

Next, we evaluated the effect of human microbial colonization on adiposity and glucose metabolism in gnotobiotic-recipient mice. Epigonadal white adipose tissue (eWAT) and spleen weights increased after colonization compared to the GF control group with the same trend in liver weights (Fig. 2A–C), despite no significant differences in feces weight, energy loss, fecal energy content, food consumption, and energy absorption during the 3 weeks following the inoculation (Fig. 2D, Supplementary Figure 2A-D). Moreover, eWAT was significantly lower in mice colonized with the CalRes compared to mice colonized with the AdLib microbiota (Fig. 2B). The reduction in body weight was associated with reduced cecum weight after colonization (Fig. 2E, Supplementary Figure 2E), which has been reported to be a hallmark of the colonization process [16, 61]. However, we did not observe significant changes in body weight gain over the course of the experiment between mice colonized with the AdLib and mice colonized with the CalRes microbiota (Supplementary Figure 2E). To further identify metabolic changes between GF and colonized mice, we performed oral glucose tolerance tests 3 weeks after the colonization. Glucose levels over time tended to be lower and glucose area under the curve was significantly lower in mice receiving the human CalRes microbiota compared to mice receiving the AdLib sample of the same donor (Fig. 2F, G). Similar to our previous work [34], these data demonstrate that caloric restriction-induced changes in the transferred human gut microbiota may induce metabolic improvement, although further experiments including dietary challenges of the recipient mice are needed to more precisely define the link between microbial and metabolic alterations.

### The CalRes microbiota reduces levels of intestinal effector memory CD8^+^ T cells and memory B cells

**Fig. 3:**
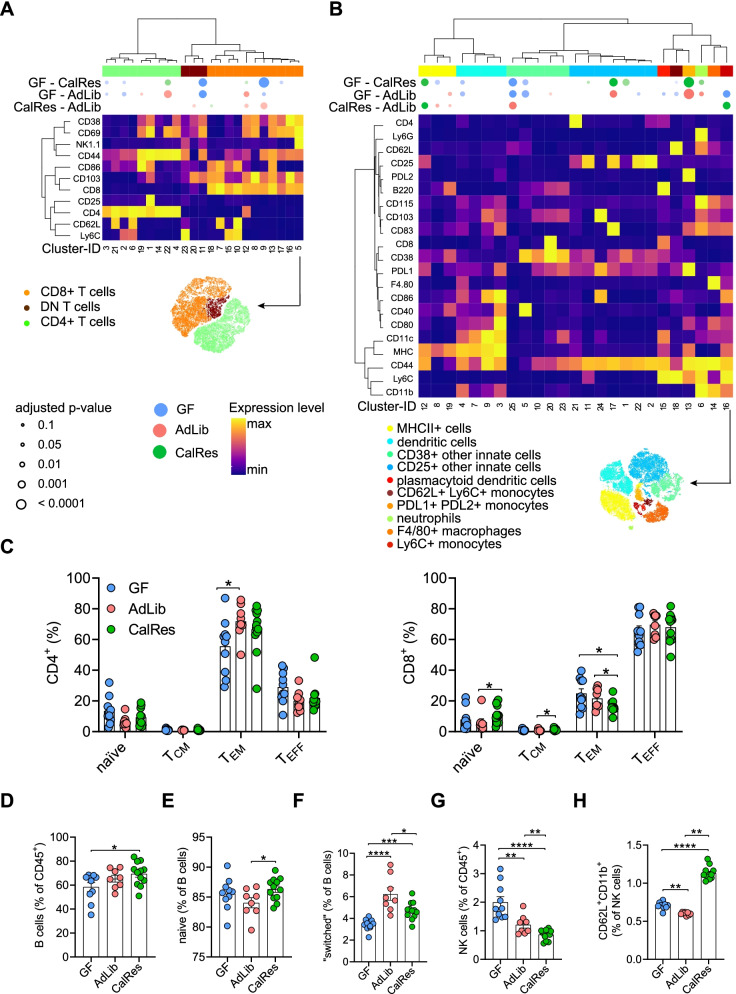
The CalRes microbiota reduces levels of intestinal effector memory CD8^+^ T cells and memory B cells. **A**, **B** The heatmaps show cluster phenotypes based on the expression of canonical lineage markers on pre-gated TCRß^+^ T cells (**A**) and on pre-gated TCRß^−^CD19^−^NK1.1^−^ innate immune cells (**B**). The differential expression of each selected surface marker (rows) is shown for each immune cell cluster (columns). The significance levels of the comparison between the three mouse groups for each immune cell cluster are depicted by semi-supervised hierarchical clustering. The top bubbles denote clusters with significantly different abundances between the three groups. Bubble colors indicate one of the two groups being compared with higher average cellular frequencies; bubble size indicates the -log2 FDR-adjusted *p*-values. **C** Relative proportions of CD4^+^ (left panel) and CD8^+^ (right panel) naïve (T_naïve_, CD44^−^CD62L^+^), central memory (T_CM_, CD44^+^CD62L^+^), effector memory (T_EM_, CD44^+^CD62L^−^) and terminally differentiated effector memory T cells (T_EMRA_, CD44^−^CD62L^−^) measured by mass cytometry. T cell populations were manually gated according to established lineage markers. **D–H** Relative proportions of total B cells (**D**), naïve B cells (**E**), “switched” memory B cells (**F**), NK cells (**G**) and activated CD62L^+^CD11b^+^ NK cells (**H**). *n* = 9 or more mice per group. * *P* < 0.05, ** *P* < 0.01, *** *P* < 0.001, **** *P* < 0.0001 as determined using Student’s *t*-test or Mann-Whitney test, dependent on the distribution of the data

Given the increasing evidence for the crosstalk between the gut microbiota and the host immune system [16], we aimed to elucidate whether the intestinal immune cell composition would differ between GF and AdLib- and CalRes-recipient mice. Using multidimensional mass cytometry and a metal-conjugated antibody panel that has been described elsewhere [43], we first analyzed colon immune cells 3 weeks post-colonization. We generated a global lineage 2D *t*-distributed neighbor embedding (*t*-SNE) map based on lineage-specific differentiation markers and performed PhenoGraph clustering after gating on viable intestinal CD45^+^ leukocytes. The unbiased comparison between the relative cellular distributions of the three mouse groups revealed that each group (AdLib, CalRes, or GF) can be almost exclusively differentiated by their immune cell profile (Supplementary Figure 3A). Next, we analyzed the distribution of innate and adaptive immune cells, using PhenoGraph clustering and t-SNE on all lineages (Supplementary Figure 3B), on pre-gated TCRß^+^ T cells (Fig. 3A), and on pre-gated TCRß^−^CD19^−^NK1.1^−^ innate immune cells (Fig. 3B). The differential expression of surface and activation markers for T cells is shown in the heatmap for each of the 23 sub-clusters. Colonization with the human microbiota led to higher proportions of CD4^+^ T cells expressing the memory and activation markers CD44 and CD69 (cluster 22, Fig. 3A). Double-negative T cells expressing CD38, CD69, and CD103 were significantly higher in GF mice (cluster 11, Fig. 3A). In the CD8^+^ T cell compartment, we observed increased levels of cells with high expression of CD44, CD69, and CD38 and low expression of CD62L (that can be defined as effector memory T cells) in mice colonized with obesity-associated AdLib compared with mice colonized with the CalRes microbiota (cluster 9, Fig. 3A). In the innate immune cell compartment, we found significantly higher levels of immune cell clusters expressing the activation markers CD40 and CD38 in GF mice (cluster 5, Fig. 3B), whereas the activation markers CD25 and CD83 were significantly higher in both colonized mouse groups (cluster 17, Fig. 3B). The proportions of myeloid cells expressing the immune checkpoint receptor ligands PD-L1 and PD-L2 and the memory marker CD44 were also significantly higher in colonized mice (cluster 13, Fig. 3B). Moreover, CalRes-recipient mice exhibited significantly higher proportions of the activation markers CD83 and CD86 compared to AdLib-recipient mice (cluster 16, Fig. 3B).

Manual gating allowed us to define known subsets and to compare levels of distinct subsets within the adaptive and innate immune cell compartment. Colonization with the AdLib microbiota increased the frequencies of effector memory CD4^+^ T cells (Fig. 3C, left panel). However, colonization with the CalRes microbiota reversed this trend in the CD8^+^ T cell compartment with increased frequencies of naïve CD8^+^ T cells, whereas the levels of effector memory CD8^+^ T cells were decreased (Fig. 3C, right panel). We found similar results in the B cell compartment with increased B cell frequencies in mice colonized with CalRes-associated microbiota (Fig. 3D), whereas the frequencies of naïve B cells were increased and the frequencies of “switched” memory B cells were decreased (Fig. 3E, F). In addition, mass cytometry quantification of absolute numbers of colonic leukocytes, CD4^+^ and CD8^+^ T cells, and B cells revealed no differences between these groups (Supplementary Figure 3C-F). Interestingly, both colonized mouse groups exhibited significantly lower frequencies of NK cells compared to GF mice, and CalRes recipients showed even lower NK cell frequencies compared to AdLib recipients (Fig. 3G). Mice colonized with the CalRes microbiota also had lower absolute numbers of NK cells compared to GF mice (Supplementary Figure 3G), and significantly higher levels of CD62L^+^ NK cells compared to the other mouse groups (Fig. 3H). These results suggest that diet-induced microbial alterations can lead to altered frequencies of effector memory T and B cells and, thus, can induce a delay of immune cell senescence in the colon.

### Colonization with human gut microbiota induces alterations of splenic immune cell subsets

**Fig. 4:**
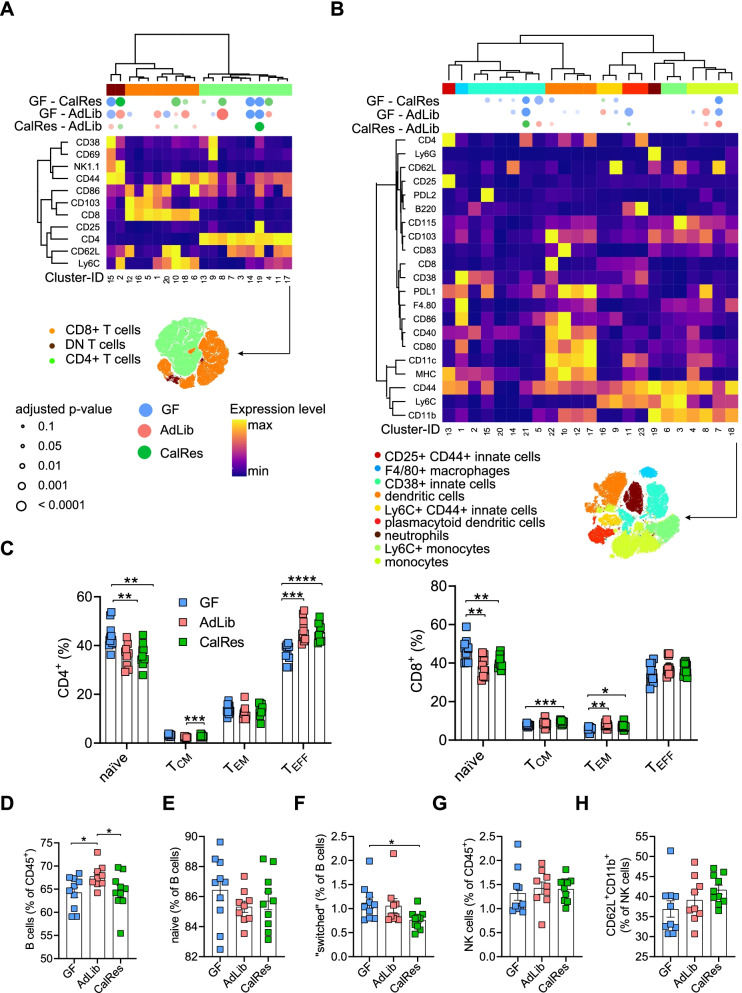
Colonization with human gut microbiota induces alterations of splenic immune cell subsets. **A**, **B** The heatmaps show the distribution of splenic immune lineages based on the expression of canonical lineage markers by t-SNE on pre-gated TCRß^+^ T cells (**A**) and on pre-gated TCRß^−^CD19^−^NK1.1^−^ innate immune cells (**B**). The differential expression of each selected surface marker (rows) is shown for each immune cell cluster (columns). The significance levels of the comparison between the three mouse groups for each immune cell cluster are depicted by semi-supervised hierarchical clustering. The top bubbles denote clusters with significantly different abundances between the three groups. Bubble colors indicate one of the two groups being compared with higher average cellular frequencies; bubble size indicates the − log2 FDR-adjusted *P*-values. **C** Relative proportions of CD4^+^ (left panel) and CD8^+^ (right panel) naïve (T_naïve_, CD44^−^CD62L^+^), central memory (T_CM_, CD44^+^CD62L^+^), effector memory (T_EM_, CD44^+^CD62L^−^) and terminally differentiated effector memory T cells (T_EMRA_, CD44^−^CD62L^−^) measured by mass cytometry. T cell populations were manually gated according to established lineage markers. **D**–**H** Relative proportions of B cells (**D**), naïve B cells (**E**), “switched” memory B cells (**F**), NK cells (**G**), and activated CD62L^+^CD11b^+^ NK cells (**H**). *n* = 9 or more mice per group. * *P* < 0.05, ** *P* < 0.01, *** *P* < 0.001, **** *P* < 0.0001 as determined using Student’s t-test or Mann-Whitney test, dependent on the distribution of the data

Given that transplantation of human feces affected by caloric restriction was sufficient to reveal differential accumulation of naïve and effector memory CD4^+^ and CD8^+^ T cells, and memory B cells in the gut compared to the obese ad libitum state, we next tested whether these differences in adaptive immune cells would also be detectable systemically and, thus, investigated immune cell subsets in the spleen. PhenoGraph clustering after gating on viable splenic CD45^+^ leukocytes revealed distinct immune cell distributions in GF mice, AdLib-recipient mice, and CalRes-recipient mice (Supplementary Figure 4A). Similar to our previous analysis of colonic immune cells, we used t-SNE on all lineages (Supplementary Figure 4B), on pre-gated splenic TCRß^+^ T cells (Fig. 4A), and on TCRß^−^CD19^−^NK1.1^−^ innate immune cells (Fig. 4B) to detect differential expression levels of cell surface markers. CD4^+^, CD8^+^, and double-negative T cells were divided into a total of 20 immune cell clusters. GF mice had significantly more abundant clusters expressing high levels of CD4, CD62L, and CD25 compared to both groups of colonized mice (cluster 19, Fig. 4A), whereas colonized mice had increased levels of clusters 18, 6, and 13 expressing T cell memory markers (CD44, Fig. 4A). CalRes-recipient mice exhibited increased proportions of a subset with high expression of CD8, CD62L, and CD44 (central memory CD8^+^ T cells) compared to mice colonized with the AdLib microbiota (cluster 10, Fig. 4A). Differences in a cluster with high expression of CD4 and CD25 could also be observed, with increased levels in mice colonized with the CalRes microbiota vs. the AdLib group (cluster 19). However, there were no significant differences in other immune cell clusters in the T cell compartment between mice colonized with the AdLib or CalRes microbiota (Fig. 4A). Interestingly, GF mice exhibited higher levels of most of the 23 clusters of the splenic myeloid compartment that expressed surface markers for macrophages and monocytes in comparison with colonized mice (clusters 21, 5 and 7, Fig. 4B). Immunophenotyping of innate immune cell populations in the spleen, did not reveal major changes comparing AdLib-recipient mice to CalRes-recipient mice. To further evaluate differences between GF and colonized mice, we analyzed innate and adaptive immune cells by manual gating. Microbiota colonization decreased the frequencies of naïve CD4^+^ and CD8^+^ T cells independent of caloric restriction but led to a significant increase of terminally differentiated effector CD4^+^ T cells and effector memory CD8^+^ T cells (Fig. 4C). As expected, absolute numbers of leukocytes, CD4^+^ T cells, CD8^+^ T cells, B cells, and NK cells were significantly increased in human gut microbiota-recipient mice (Supplementary Figure 4C-G). There were no significant changes in the splenic T cell compartment between mice colonized with the AdLib and mice colonized with the CalRes microbiota. However, we observed a significant decrease of total B cell frequencies in CalRes-recipient mice compared to AdLib-recipient mice and a slight decrease of “switched” memory B cell frequencies within the B cell compartment in mice colonized with the CalRes human gut microbiota compared to GF mice (Fig. 4D–F). We found no differences in splenic NK cell frequencies or their activation state (Fig. 4G, H). Our results suggest that colonization with the human gut microbiota into GF mice led to significant alterations of systemic innate and adaptive immune cells.

### Caloric restriction-associated gut microbiota reduces levels of hepatic effector memory CD4^+^ and CD8^+^ T cells

**Fig. 5:**
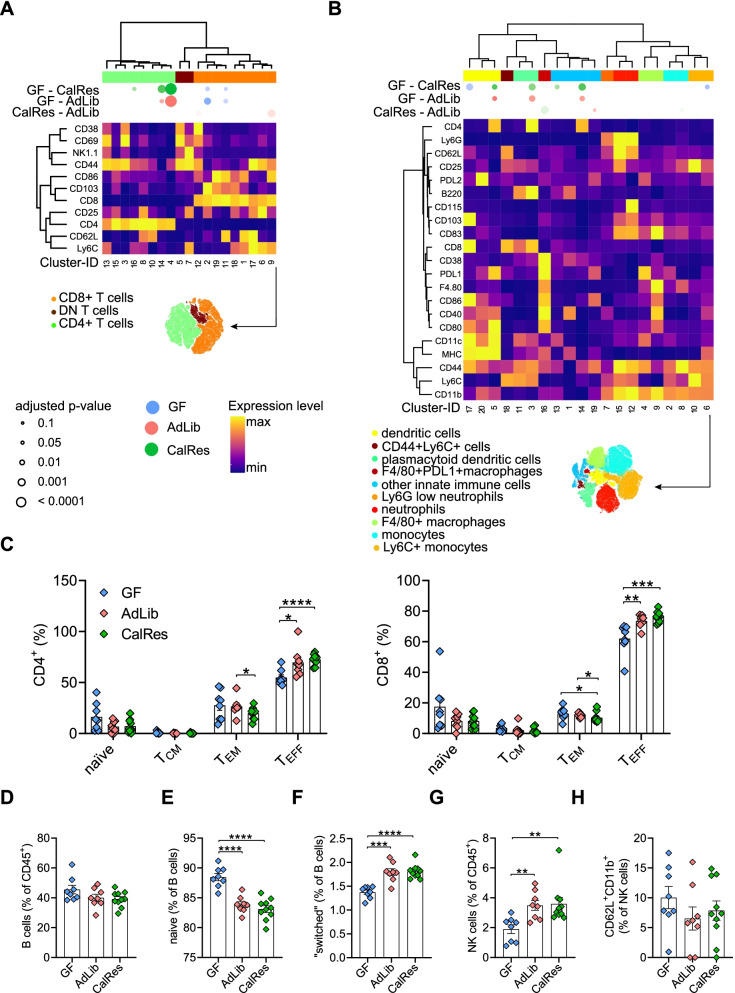
Caloric restriction-associated gut microbiota reduces levels of hepatic effector memory CD4^+^ and CD8^+^ T cells. **A**, **B** The heatmaps show the distribution of hepatic immune lineages based on the expression of canonical lineage markers by t-SNE on pre-gated TCRß^+^ T cells (**A**) and on pre-gated TCRß^−^CD19^−^NK1.1^−^ innate immune cells (**B**). The differential expression of each selected surface marker (rows) is shown for each immune cell cluster (columns). The significance levels of the comparison between the three mouse groups for each immune cell cluster are depicted by semi-supervised hierarchical clustering. The top bubbles denote clusters with significantly different abundances between the three groups. Bubble colors indicate the one of the two groups being compared with higher average cellular frequencies; bubble size indicates the − log2 FDR-adjusted *p*-values. **C** Relative proportions of CD4^+^ (left panel) and CD8^+^ (right panel) naïve (T_naïve_, CD44^−^CD62L^+^), central memory (T_CM_, CD44^+^CD62L^+^), effector memory (T_EM_, CD44^+^CD62L^−^) and terminally differentiated effector memory T cells (T_EMRA_, CD44^−^CD62L^−^) measured by mass cytometry. T cell populations were manually gated according to established lineage markers. **D–H** Relative proportions of B cells (**D**), naïve B cells (**E**), “switched” memory B cells (**F**), NK cells (**G**), and activated CD62L^+^CD11b^+^ NK cells (**H**). *n* = 9 or more mice per group. * *P* < 0.05, ** *P* < 0.01, *** *P* < 0.001, **** *P* < 0.0001 as determined using Student’s *t*-test or Mann-Whitney test, dependent on the distribution of the data

We next set out to elucidate whether diet-induced changes within the human gut microbiota would translate to differences in hepatic immune cell changes in the gnotobiotic host. Recent studies on the colonization-induced host-gut microbial interaction revealed that the colonization process stimulated glycogenesis in the liver, increased hepatic triglyceride synthesis, and that the microbial composition was strongly associated with hepatic metabolic profiles [62], raising the question whether the hepatic immune cell composition was also affected. We first addressed this question using PhenoGraph clustering after gating on viable hepatic CD45^+^ leukocytes, which revealed distinct immune cell distributions in GF mice, AdLib-recipient mice, and CalRes-recipient mice, similar to our observations in colon and spleen (Supplementary Figure 5A). Next, we used PhenoGraph clustering on all lineages (Supplementary Figure 5B), on pre-gated splenic TCRß^+^ T cells (Fig. 5A), and TCRß^−^CD19^−^NK1.1^−^ innate immune cells (Fig. 5B), as described above. A total of 19 clusters were defined in the T cell compartment of murine livers, whereas the myeloid compartment consisted of a total of 20 clusters. Microbial colonization increased levels of hepatic CD4^+^ T cell clusters expressing the memory marker CD44 (cluster 4, Fig. 5A), consistent with previous findings in the colon and spleen. Moreover, the frequencies of clusters comprising naïve CD8^+^ T cells (high expression of CD62L and low expression of CD44) were significantly higher in GF mice compared to mice inoculated with the human gut microbiota (cluster 11, Fig. 5A). Similarly, microbial colonization increased levels of CD4^+^ expressing innate immune cell clusters, which also exhibited expression of activation markers, such as CD80 and CD40 (cluster 5, Fig. 5B). Comparing levels of distinct hepatic T cell subsets between the three mouse groups by manual gating, we observed a significant drop in effector memory CD4^+^ T cells in mice colonized with the CalRes microbiota, whereas effector CD4^+^ T cells were increased in both colonized mouse groups compared with GF mice, with a similar pattern in the CD8^+^ T cell compartment (Fig. 5C). In line with our previous findings in the colon and spleen, microbial colonization led to significantly decreased levels of naive B cells in favor of increased levels of “switched” memory B cells (Fig. 5D–F). In contrast to our findings in the colon, the frequency of hepatic NK cells was increased in colonized mice, whereas no significant changes in the activation state of NK cells were observed (Fig. 5G, H). Absolute numbers of leukocytes, CD4^+^ T cells, CD8^+^ T cells, B cells, and NK cells were similar between the three groups (Supplementary Figure 5C-G). Together, these results suggest that the CalRes microbiota not only induced alterations in frequencies of memory cells in the colon, but similarly led to delayed T cell senescence in the liver.

### Immune cell composition correlates with microbial taxa

**Fig. 6:**
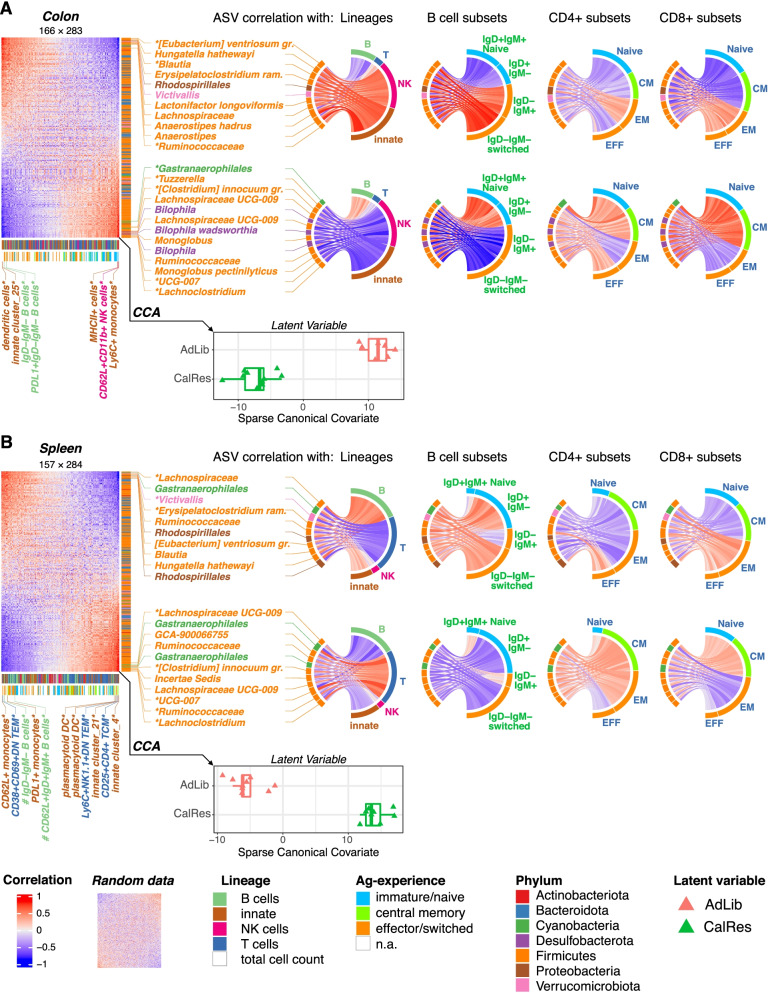
Immunologic changes correlate with gut microbial alterations. Heatmaps show latent correlation matrices between abundances of amplicon sequence variants (ASVs) detected in stool samples and all immune parameters analyzed in colon (**A**) and spleen (**B**) of mice 21 days after inoculation with AdLib and CalRes human gut microbiota. Immune parameters are expressed as frequencies, i.e., percent of parent, except those labeled # which were quantified as absolute cell counts. Heatmaps were ordered according to rows and columns first principal components to highlight cross-correlation structures. Asterisks indicate variables that were selected in L1-penalized sparse canonical correlation analysis (CCA). Circular chord plots display latent correlation between frequencies of manually defined immune subsets and L1-selected ASVs including the top ten taxa that either positively (upper) or negatively (lower) associate with the immunological dataset. Blue to red color scale in heatmaps and chords indicates negative and positive correlation values. Color of row-legend bar and species labels denotes the phylum level. Colors of column legend bars indicate parental lineage and differentiation level (antigen-experience) of lymphocyte subsets, respectively. Boxplot insets show how experimental groups as a latent variable are explained by the canonical covariate

To identify significant correlations between immune cell subsets across multiple organs (colon, spleen, liver) and gut microbial alterations, we used sparse canonical correlation analyses. Interestingly, microbial taxa with increased abundances in mice colonized with the CalRes microbiota, including *M. pectinilyticus*, *Tuzzerella*, or *Lachnospiraceae UCG-009* were associated with a decreased percentage of NK cells and other innate immune cells such as dendritic cells and macrophages in the colon (Fig. 6A), in which the levels of innate immune cells are dominant compared to B and T cells. In line with these data, NK cell frequencies were recently shown to be reduced in peripheral tissues and to exhibit an altered phenotype in the spleen in mice that underwent caloric restriction [63]. CalRes-associated taxa were also positively associated with intestinal naïve and central memory CD4^+^ and CD8^+^ T and naïve B cell subsets, whereas taxa with high abundance in mice colonized with the AdLib microbiota such as *E. ventriosum gr.*, *H. hathewayi*, *E*. *ramosum*, and *Rhodospirillales* correlated positively with switched B cell and memory/effector T cell subsets in the colon (Fig. 6A). In addition, we found positive associations between *E. ventriosum gr.*, *H. hathewayi*, *E*. *ramosum*, and *Rhodospirillales* with percentages of splenic switched B cells and memory/effector CD4^+^ and CD8^+^ T cells, whereas taxa with high abundance in the CalRes group, including *Lachnospiraceae* and *Ruminococcaceae* correlated positively with naïve and central memory CD4^+^ and CD8^+^ T cells in the spleen (Fig. 6B). Similar results were observed in the liver, where taxa belonging to the phylum Firmicutes were positively associated with CD44^−^CD62L^−^ effector CD4^+^ and CD8^+^ T cells, whereas taxa with high abundance in the CalRes group correlated positively with naïve B and naïve and central memory T cell subsets (Supplementary Figure 6). These findings highlight the impact of caloric restriction on alterations in gut microbial communities, which may subsequently attenuate immune senescence across multiple organs.

## Discussion

Obesity is linked to alterations in the human gut microbiome and chronic low-grade inflammation, potentially affecting numerous metabolic pathways including the development of fatty liver disease and insulin resistance. Caloric restriction induces distinct metabolic alterations in host metabolism and may play an important role by influencing the activation and effector functions of immune cells. Here, we used multiparameter mass cytometry combined with a clinical diet intervention in humans and 16S rRNA sequencing of the recipient gut microbiome to test whether diet-induced changes in the human gut microbiome would affect the host’s immune response. Transplantation of fecal samples from the obese human donor before (AdLib) and after (CalRes) caloric restriction-induced maturation of distinct microbial communities in gnotobiotic-recipient mice. The initial drop in body weight after colonization, as well as increased adiposity, epigonadal fat expansion and impaired glucose tolerance in the delayed phase after colonization of GF mice has already been reported [16] and is consistent with our findings. Multiple studies have shown that switching from a high-fat, high-sugar “Western” diet to caloric restriction can shift the structure of microbial communities and alter microbiome gene expression rapidly [28, 31, 32]. We observed a reduction in several taxa in the CalRes-recipient mice, including *B. vulgatus*, *Eubacterium ventriosum group*, *Dorea*, *Adlercreutzia aequolifaciens*, *Butyricicoccus*, *Erysipelatoclostridium ramosum*, *Anaerostipes hadrus*, *Alistipes obesi*, *Hungatella hathewayi*, *Rhodospirillales*, and *Bacteroides uniformis*, and increased abundances of taxa such as *Roseburia hominis*, *Bilophila wadsworthia* and *Coprococcus* consistent with previously reported taxonomic changes in our donor individual and in multiple other studies [34, 54, 64–67], with the exception of *Anaerostipes hadrus*, which has been described to increase during human weight loss [68], and *Dorea*, for which increased and decreased abundances during weight loss were reported [69–71]. Similarly, microbial colonization with the CalRes microbiota increased the abundance of taxa belonging to the phylum Bacteroidetes, whereas several bacteria belonging to the Proteobacteria family were decreased compared to mice inoculated with the AdLib microbiota. The AdLib microbiota in the gnotobiotic-recipient mice was characterized by increased abundances of taxa such as *E. ventriosum gr.*, *H. hathewayi*, *E. ramosum*, and *Rhodospirillales* which were associated with higher percentages of memory B cells and memory/effector CD4^+^ and CD8^+^ T cells in the colon and the spleen. *H. hathewayi*, *E. ramosum*, *Rhodospirillales*, and *E.ventriosum* are linked to obesity and obesity-related pathologies [51–53]. Moreover, previous clinical studies investigating the role of *H. hathewayi* have highlighted its association with colorectal cancer [72, 73] and poor response to immunotherapy in melanoma and renal cell cancer patients [74, 75]. *E. ramosum* was shown to promote high fat diet-induced obesity in gnotobiotic mice, is associated with the metabolic syndrome in humans [53], increased in Crohn’s disease, and induced Th1 cells and intestinal inflammation in gnotobiotic mice [76]. In contrast, caloric restriction increased the abundance of *M. pectinilyticus*, associated with a high intake of dietary fiber and pectin [58]. Multiple taxa belonging to the phylum *Lachnospiracae* had a significantly higher abundance in the CalRes group as well and were positively correlated with percentages of naïve B and T cells. Importantly, taxonomic differences between experimental groups are partially driven by the fact that some taxa were below the detection limit in one group or the other (Fig. 1C). Thus, we anticipate that the AdLib and CalRes communities would not further converge over time (if entirely absent), albeit being on the same diet, which is shown in Fig. 1B where community structure remained stable within each group but separated between groups after day 14.

Fecal transplantation with the human microbiota led to significant differences in naïve and memory/effector B and T cell subsets, NK cells and various other subsets of innate immune cells depending on the nutritional state of the donor. Colonization of GF mice with the AdLib human gut microbiota raised frequencies of memory/effector CD4^+^ and CD8^+^ T cell subsets, as well as frequencies of class-switched memory B cells throughout all investigated tissues. Contrastingly, the levels of effector memory CD8^+^ T cell subsets were significantly lower in mice colonized with the CalRes microbiota compared to mice colonized with the AdLib microbiota in the large intestine and the liver. Thus, the caloric restriction-associated microbiota delayed immune cell senescence, which may have initially been induced by an increase in intestinal permeability of pro-inflammatory molecules and increased hepatic inflammation in the early phase of colonization [16]. Similarly, caloric restriction preserved a naïve T cell phenotype and an immature NK cell phenotype in aged wildtype mice [15].

Consistent with our prior work, mice inoculated with the AdLib gut microbiota exhibited increased epigonadal fat pads and lower glucose clearance during an OGTT compared to mice inoculated with the CalRes gut microbiota [34]. Whereas caloric restriction and weight loss improved systemic insulin sensitivity in human participants of our weight loss intervention study, human subcutaneous adipose tissue displayed no significant improvement of inflammatory parameters (cytokine levels and leukocyte subpopulations) and no significant changes in systemic immune cell populations could be detected [11]. Contrastingly, the CalRes microbiota induced significant alterations in memory cell subsets and attenuated immune senescence in gnotobiotic recipients maintained on a chow diet. We hypothesize that obesity-induced immunologic changes, e.g., T cell senescence, in adipose tissues could have a long-standing effect that may be hard to overcome by short-term caloric restriction-induced weight loss. Taken together, caloric restriction has the potential to reduce obesity-associated microbial taxa that are linked with systemic inflammation, cancer development and metabolic disease and subsequently attenuate immune senescence and low-grade inflammation.

Our study has several limitations. First, since the human donor samples were derived from a single individual, the results may not be generalizable to other individuals. Thus, immune responses of recipient hosts may vary with regard to individual variation of donor microbial community dynamics undergoing weight loss with VLCD, specifically if the diets vary in caloric content, macro- and/or micronutrient composition. To address this limitation, we compared the diversity dynamics of our donor samples to the original study cohort from which this donor was selected undergoing the same intervention with the same diet regime as previously reported [34]. Compared to other obese/overweight individuals in this cohort, diversity distance metrics revealed the baseline (AdLib) and diet samples (CalRes) of our individual donor (MMSP50) were representative of subjects on the diet regime. Nonetheless, the variability of host immune response to the CalRes microbiome of several individuals on cohort level remains to be determined but is limited to the constraints of the gnotobiotic experimental setting. Together with corresponding taxonomic changes during weight loss in the literature as stated above, the observed taxonomic changes during caloric restriction are fairly representative regarding known weight loss-associated community dynamics. Further, the limited transfer rate from humans to mice specifically in the Firmicutes clade [34, 77], allows only limited interpretation comparing taxonomic changes of the donor and recipients. Second, since we have used a translational approach from humans to GF mice, we can only provide indirect evidence regarding the immune response, which may be different in the human host. Whereas correlation analyses revealed significant associations between taxa that were dominant in the CalRes microbiota with naïve B and T cell subsets, only monocolonization of GF mice with specific bacterial species followed by systematic analyzation of host immunologic adaptation to colonization would provide a causal evaluation of the effect of individual bacterial species on the composition and activation of innate and adaptive immune cells [27]. Third, mice were not singly housed but in groups of two to four mice per cage (three cages per experimental group, respectively). We observed a skewed abundance distribution for a few taxa as shown by leaflet numbers in Fig. 1C (*Colidextribacter*, *Lachnospiraceae NK4A136 group*, and *Coprococcus comes* were more abundant in cage 1 of the CalRes groups, *Lachnospiraceae* were more abundant in cages 2 and 3 of the AdLib group) which needs to be taken into consideration for interpretation of the data. However, the abundance distribution of the most taxa was even between cages of each experimental group. Another caveat is that amplicon sequencing frequently lacks the resolution to resolve species and strains which may be phenotypically diverse in their host-microbe interactions [78].

Key questions regarding the mechanisms that are mediating the crosstalk between microbial communities, tissue immune cell composition, and metabolism in both mice and humans, and the underlying immune signaling pathways remain open. Nevertheless, this work extends previous important findings on the microbiota-host immune system crosstalk [26, 27], by studying the interaction of the obesity- and caloric restriction-associated human gut microbiota on immune responses in several murine tissues via multiparameter mass cytometry. We validated the computational unbiased analysis of our multidimensional mass cytometry data by manual gating and were able to show that both methods led to similar results, as shown before [43]. Additionally, future studies should address the question whether these effects are long-lasting or highly dependent on immediate changes in the diet. Another critical question is if diet-induced changes within the microbiome of children may exert similar or divergent effects on immune cell maturation.

## Conclusion

Taken together, we provide first data for a better understanding of the host immune system-microbiota interaction in the context of caloric restriction which may impact metabolic diseases such as obesity and type 2 diabetes.

## Supplementary Information

**Additional file 1: Supplementary Figure 1.** Related to Fig. 1. MMSP50 is a representative donor of the weight loss cohort. **A** Examination of baseline alpha diversity demonstrates that MMSP50 is at the 54^th^ ranked percentile for baseline diversity after VLCD. **B** Their baseline microbiota composition (principal coordinates analysis of Bray-Curtis Dissimilarity) is well within the 95% confidence interval of baseline composition for the cohort (dotted line) and **C** their change in community structure is the 19^th^ percentile for change in composition. **Supplementary Figure 2.** Related to Fig. 2. No significant changes in energy loss or fecal content after microbial colonization. Metabolic analysis of germ-free (GF) mice and mice inoculated with the AdLib and CalRes human gut microbiota. **A-D** Energy loss (A), fecal energy content (B), food consumption (C), and energy absorption (D) were measured using bomb calorimetry in GF and colonized mice. **E** Body weights in g. ** *P* < 0.01, *** *P* < 0.001 as determined using 2-way ANOVA with Bonferonni’s post-test correction for multiple comparisons. error bars = SEM. **Supplementary Figure 3.** Related to Fig. 3. Differential expression of surface markers in different colonic immune cell clusters of germ-free and colonized mice. **A** The heatmap shows differentially distributed colonic immune cell phenotypes quantified by PhenoGraph clustering. The distribution of each cell cluster (rows) is shown for each murine sample (columns). **B** The heatmap shows the distribution of colonic immune lineages based on the expression of canonical lineage markers by t-SNE on all colonic viable CD45^+^ leukocytes. The differential expression of each selected surface marker (rows) is shown for each immune cell cluster (columns). The significance levels of the comparison between the groups for each immune cell cluster are depicted by semi-supervised hierarchical clustering. The top bubbles denote clusters with significantly different abundances between the groups. Bubble colors indicate the one of the two groups being compared with higher average cellular frequencies; bubble size indicates the -log2 FDR-adjusted *p*-values. Visualization of all colonic viable CD45^+^ leukocytes by t-SNE. Overlayed colors represent Phenograph clusters as defined in heatmap. **C-G** Absolute numbers of colonic leukocytes (C), CD4^+^ T cells (D), CD8^+^ T cells (E), B cells (F), and NK cells (G) defined by manual gating of mass cytometry data, from germ-free (GF) mice and mice colonized with the AdLib and CalRes human gut microbiota from the top weight loser of an 8-week weight loss intervention study (*n*=9 or more mice per group). * *P*<0.05, ANOVA with Bonferonni’s post-test correction for multiple comparison. **Supplementary Figure 4.** Related to Fig. 4. Differential expression of surface markers in different splenic immune cell clusters of germ-free and colonized mice. **A** The heatmap shows differentially distributed splenic immune cell phenotypes quantified by PhenoGraph clustering. The distribution of each cell cluster (rows) is shown for each murine sample (columns). **B** The heatmap shows the distribution of splenic immune lineages based on the expression of canonical lineage markers by t-SNE on all colonic viable CD45^+^ leukocytes. The differential expression of each selected surface marker (rows) is shown for each immune cell cluster (columns). The significance levels of the comparison between the groups for each immune cell cluster are depicted by semi-supervised hierarchical clustering. The top bubbles denote clusters with significantly different abundances between the groups. Bubble colors indicate the one of the two groups being compared with higher average cellular frequencies; bubble size indicates the -log2 FDR-adjusted *p*-values. Visualization of all splenic viable CD45^+^ leukocytes by t-SNE. Overlayed colors represent Phenograph clusters as defined in heatmap. **C-G** Absolute numbers of splenic leukocytes (C), CD4^+^ T cells (D), CD8^+^ T cells (E), B cells (F), and NK cells (G) defined by manual gating of mass cytometry data, from germ-free (GF) mice and mice colonized with the AdLib and CalRes human gut microbiota from the top weight loser of an 8-week weight loss intervention study (*n*=9 or more mice per group). * P<0.05, ANOVA with Bonferonni’s post-test correction for multiple comparison. **Supplementary Figure 5.** Related to Fig. 5. Differential expression of surface markers in different hepatic immune cell clusters of germ-free and colonized mice. **A** The heatmap shows differentially distributed hepatic immune cell phenotypes quantified by PhenoGraph clustering. The distribution of each cell cluster (rows) is shown for each murine sample (columns). **B** The heatmap shows the distribution of hepatic immune lineages based on the expression of canonical lineage markers by t-SNE on all colonic viable CD45^+^ leukocytes. The differential expression of each selected surface marker (rows) is shown for each immune cell cluster (columns). The significance levels of the comparison between the groups for each immune cell cluster are depicted by semi-supervised hierarchical clustering. The top bubbles denote clusters with significantly different abundances between the groups. Bubble colors indicate the one of the two groups being compared with higher average cellular frequencies; bubble size indicates the -log2 FDR-adjusted p-values. Visualization of all hepatic viable CD45^+^ leukocytes by t-SNE. Overlayed colors represent Phenograph clusters as defined in heatmap. **C-G** Absolute numbers of hepatic leukocytes (C), CD4^+^ T cells (D), CD8^+^ T cells (E), B cells (F), and NK cells (G) defined by manual gating of mass cytometry data, from germ-free (GF) mice and mice colonized with the AdLib and CalRes human gut microbiota from the top weight loser of an 8-week weight loss intervention study (*n*=9 or more mice per group). ANOVA with Bonferonni’s post-test correction for multiple comparison. **Supplementary Figure 6.** Related to Fig. 6. Gut microbial community structure slightly affects composition and activation of liver immune cells. The heatmap shows latent correlation matrix between abundances of amplicon sequence variants (ASVs) detected in stool samples and all immune parameters analyzed in liver of mice 21 days after inoculation with AdLib and CalRes human gut microbiota. Immune parameters are expressed as frequencies, i.e., percent of parent, except those labeled # which were quantified as absolute cell counts. Heatmap was ordered according to rows and columns first principal components to highlight the cross-correlation structure. Asterisks indicate variables that were selected in L1-penalized sparse canonical correlation analysis (CCA). Circular chord plots display latent correlation between frequencies of manually defined immune subsets and L1-selected ASVs including the top ten taxa that either positively or negatively associate with the immunological dataset. Blue to red colour scale in heatmap and chords indicates negative and positive correlation values. Color of row-legend bar and species labels denotes the phylum level. Colors of column legend bars indicate parental lineage and differentiation level (antigen-experience) of lymphocyte subsets, respectively. The boxplot inset shows how experimental groups as a latent variable are not well-explained by the sparse canonical covariate.

**Additional file 2. **The three supplementary Excel files, for colon (colon_cca_xlsx), spleen (spleen_cca_xlsx), and liver(liver_cca_xlsx), comprise each four spreadsheets: The sheets “corr.matrix” and “corr.fdr” contain estimated coefficients of the latent cross-correlation matrix (as shown in Fig. 6 and Supplementary Figure 5 heatmaps) and corresponding FDR-adjusted *P*-values, respectively. The sheet “asv.cca” contains for all ASVs (with the same order as in the correlation matrix) FDR-adjusted *P*-values and log-fold change values (columns C, D) from DESeq2 differential abundance testing (as shown in heatmap Fig. 1 C), the corresponding taxonomy table including seven taxonomic ranks (columns E-K), taxa labels used for heatmaps (column L) and two columns indicating the top (upper and lower) ASVs that were highlighted in correlation heatmap and circular chord diagrams (column M) and that were selected by sparse canonical correlation analysis using the Bayesian information criterion (column N), with sparse coefficients’ weights provided in column O. The sheet “imm.cca” contains for all immunological parameters (with the same order as in the correlation matrix) that were assessed in a given organ, FDR-adjusted *P*-values from GLMM and non-parametric (Wilcoxon-Mann-Whitney) differential abundance testing (columns C, D) between AdLib and CalRes groups. Columns E indicates type of parameter, i.e., whether absolute cell counts, or cell frequencies were determined. Column F denotes which cell population served as input for cluster-based analyses or was manually gated. Columns G – I provide cell subset IDs including prepended cluster numbers, manually annotated population names, and differentiation state. Columns J – L contain labels as displayed in correlation heatmap and circular chord diagrams for subset name, differentiation/antigen-experience, and lineage membership, respectively. Columns M – O indicate the top left and right immune parameters in the correlation heatmap, those selected by sparse canonical correlation analysis using the Bayesian information criterion, and sparse coefficients’ weights, respectively.

## Data Availability

Sequence files and metadata for the 16SrRNA seq analyses for all samples used in this study have been deposited in Figshare (https://figshare.com/s/42295e6a08511c63a870). This link includes the R script to reproduce the statistical analyses and generation of Figs. 1 and 6 and Supplementary Figure 5.

## Abbreviations

Ad Lib: Before 8-week caloric restriction program
CalRes: After 8-week caloric restriction program
FMT: Fecal microbiota transfer
GF: Germ-free
GLMM: Generalized linear mixed-effects model
IL-6: Interleukin-6
Th1: CD4^+^-type 1 helper T cells
Th17: CD4^+^-type 3/17 helper T cells
VLCD: Very low-calorie diet
