# Supplementary figures and images for: Effects of caloric restriction on the gut microbiome are linked with immune senescence
Source: Microbiome. 2022 Apr 4;10:57. doi: 10.1186/s40168-022-01249-4 (PMC8978410; doi:10.1186/s40168-022-01249-4)

**A**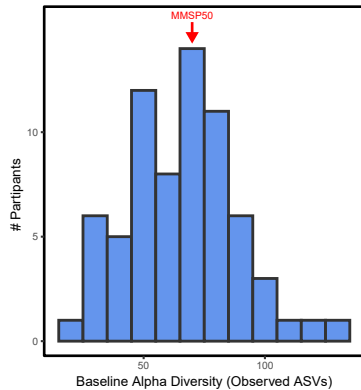**B**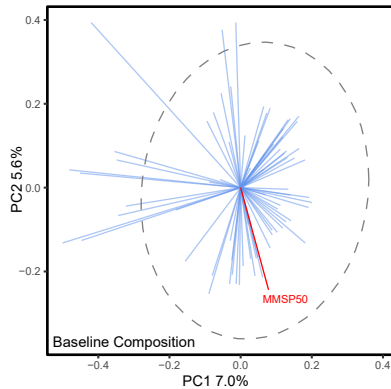**C**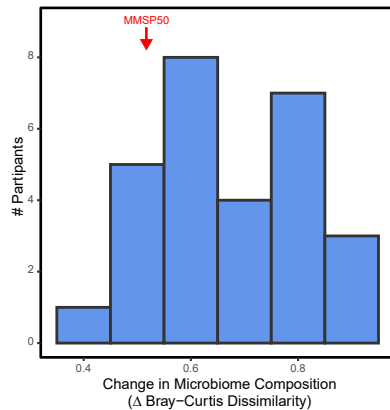

Supplement: Supplementary file 2 — Additional file 1: Supplementary Figure 1. Related to Fig. 1. MMSP50 is a representative donor of the weight loss cohort. A Examination of baseline alpha diversity demonstrates that MMSP50 is at the 54th ranked percentile for baseline diversity after VLCD. B Their baseline microbiota composition (principal coordinates analysis of Bray-Curtis Dissimilarity) is well within the 95% confidence interval of baseline composition for the cohort (dotted line) and C their change in community structure is the 19th percentile for change in composition. Supplementary Figure 2. Related to Fig. 2. No significant changes in energy loss or fecal content after microbial colonization. Metabolic analysis of germ-free (GF) mice and mice inoculated with the AdLib and CalRes human gut microbiota. A-D Energy loss (A), fecal energy content (B), food consumption (C), and energy absorption (D) were measured using bomb calorimetry in GF and colonized mice. E Body weights in g. ** P < 0.01, *** P < 0.001 as determined using 2-way ANOVA with Bonferonni’s post-test correction for multiple comparisons. error bars = SEM. Supplementary Figure 3. Related to Fig. 3. Differential expression of surface markers in different colonic immune cell clusters of germ-free and colonized mice. A The heatmap shows differentially distributed colonic immune cell phenotypes quantified by PhenoGraph clustering. The distribution of each cell cluster (rows) is shown for each murine sample (columns). B The heatmap shows the distribution of colonic immune lineages based on the expression of canonical lineage markers by t-SNE on all colonic viable CD45+ leukocytes. The differential expression of each selected surface marker (rows) is shown for each immune cell cluster (columns). The significance levels of the comparison between the groups for each immune cell cluster are depicted by semi-supervised hierarchical clustering. The top bubbles denote clusters with significantly different abundances between the groups. Bubble co [file 40168_2022_1249_MOESM2_ESM.zip › Supplementary Figure 1.pdf]

**A**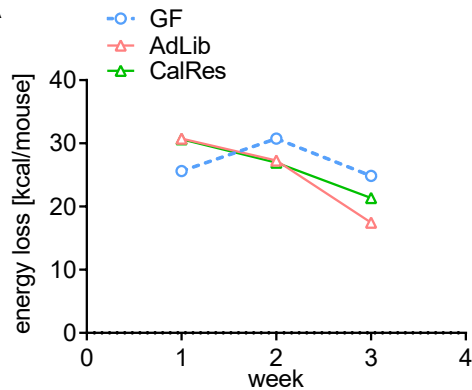**B**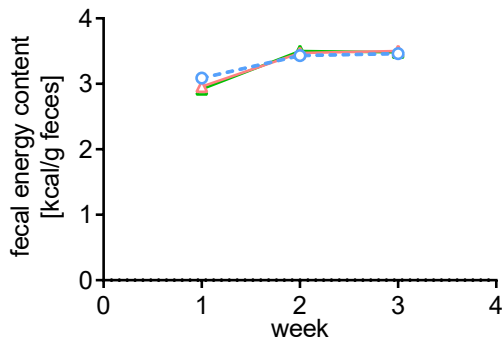**C**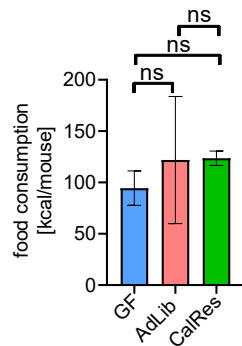**D**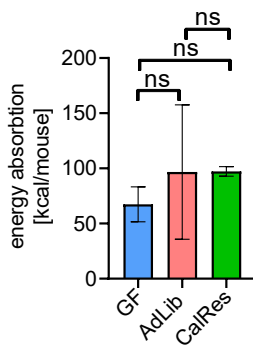**E**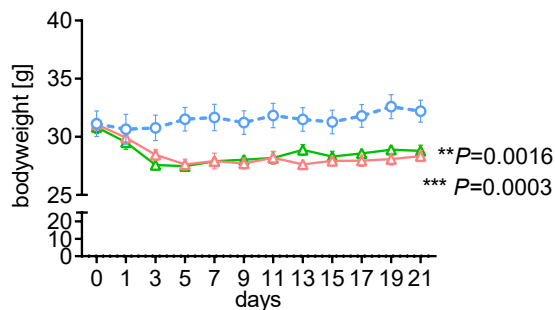

Supplement: Supplementary file 2 — Additional file 1: Supplementary Figure 1. Related to Fig. 1. MMSP50 is a representative donor of the weight loss cohort. A Examination of baseline alpha diversity demonstrates that MMSP50 is at the 54th ranked percentile for baseline diversity after VLCD. B Their baseline microbiota composition (principal coordinates analysis of Bray-Curtis Dissimilarity) is well within the 95% confidence interval of baseline composition for the cohort (dotted line) and C their change in community structure is the 19th percentile for change in composition. Supplementary Figure 2. Related to Fig. 2. No significant changes in energy loss or fecal content after microbial colonization. Metabolic analysis of germ-free (GF) mice and mice inoculated with the AdLib and CalRes human gut microbiota. A-D Energy loss (A), fecal energy content (B), food consumption (C), and energy absorption (D) were measured using bomb calorimetry in GF and colonized mice. E Body weights in g. ** P < 0.01, *** P < 0.001 as determined using 2-way ANOVA with Bonferonni’s post-test correction for multiple comparisons. error bars = SEM. Supplementary Figure 3. Related to Fig. 3. Differential expression of surface markers in different colonic immune cell clusters of germ-free and colonized mice. A The heatmap shows differentially distributed colonic immune cell phenotypes quantified by PhenoGraph clustering. The distribution of each cell cluster (rows) is shown for each murine sample (columns). B The heatmap shows the distribution of colonic immune lineages based on the expression of canonical lineage markers by t-SNE on all colonic viable CD45+ leukocytes. The differential expression of each selected surface marker (rows) is shown for each immune cell cluster (columns). The significance levels of the comparison between the groups for each immune cell cluster are depicted by semi-supervised hierarchical clustering. The top bubbles denote clusters with significantly different abundances between the groups. Bubble co [file 40168_2022_1249_MOESM2_ESM.zip › Supplementary Figure 2.pdf]

**A**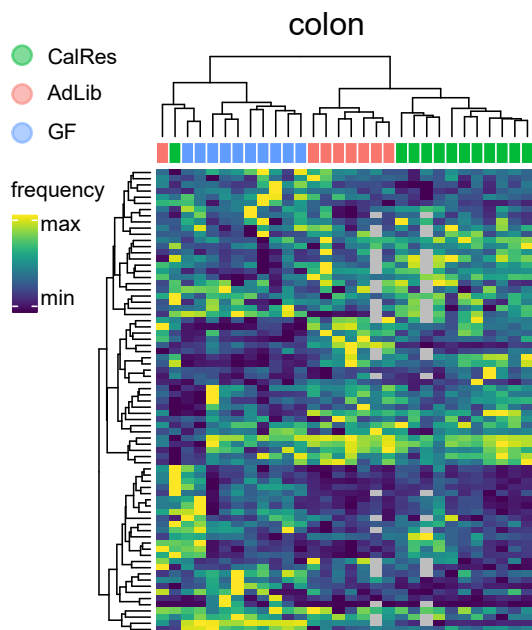**B**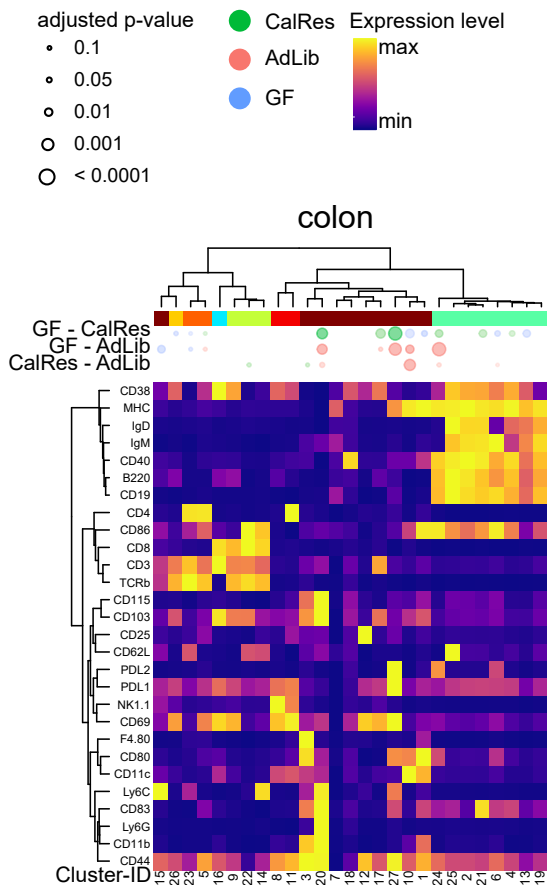**C**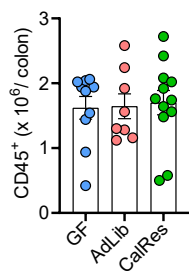**D**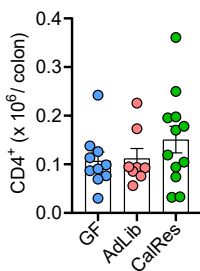**E**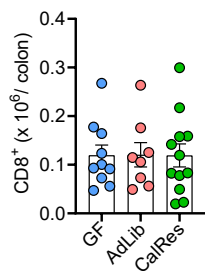**F**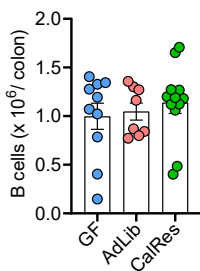**G**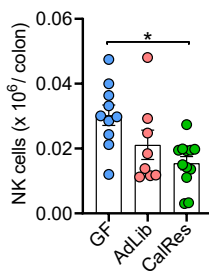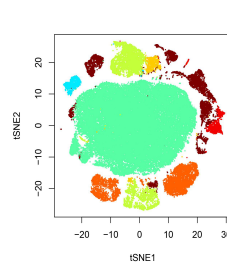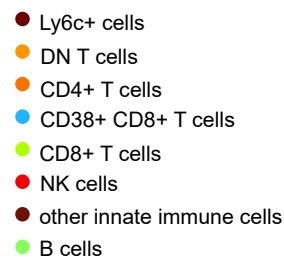

Supplement: Supplementary file 2 — Additional file 1: Supplementary Figure 1. Related to Fig. 1. MMSP50 is a representative donor of the weight loss cohort. A Examination of baseline alpha diversity demonstrates that MMSP50 is at the 54th ranked percentile for baseline diversity after VLCD. B Their baseline microbiota composition (principal coordinates analysis of Bray-Curtis Dissimilarity) is well within the 95% confidence interval of baseline composition for the cohort (dotted line) and C their change in community structure is the 19th percentile for change in composition. Supplementary Figure 2. Related to Fig. 2. No significant changes in energy loss or fecal content after microbial colonization. Metabolic analysis of germ-free (GF) mice and mice inoculated with the AdLib and CalRes human gut microbiota. A-D Energy loss (A), fecal energy content (B), food consumption (C), and energy absorption (D) were measured using bomb calorimetry in GF and colonized mice. E Body weights in g. ** P < 0.01, *** P < 0.001 as determined using 2-way ANOVA with Bonferonni’s post-test correction for multiple comparisons. error bars = SEM. Supplementary Figure 3. Related to Fig. 3. Differential expression of surface markers in different colonic immune cell clusters of germ-free and colonized mice. A The heatmap shows differentially distributed colonic immune cell phenotypes quantified by PhenoGraph clustering. The distribution of each cell cluster (rows) is shown for each murine sample (columns). B The heatmap shows the distribution of colonic immune lineages based on the expression of canonical lineage markers by t-SNE on all colonic viable CD45+ leukocytes. The differential expression of each selected surface marker (rows) is shown for each immune cell cluster (columns). The significance levels of the comparison between the groups for each immune cell cluster are depicted by semi-supervised hierarchical clustering. The top bubbles denote clusters with significantly different abundances between the groups. Bubble co [file 40168_2022_1249_MOESM2_ESM.zip › Supplementary Figure 3.pdf]

**A**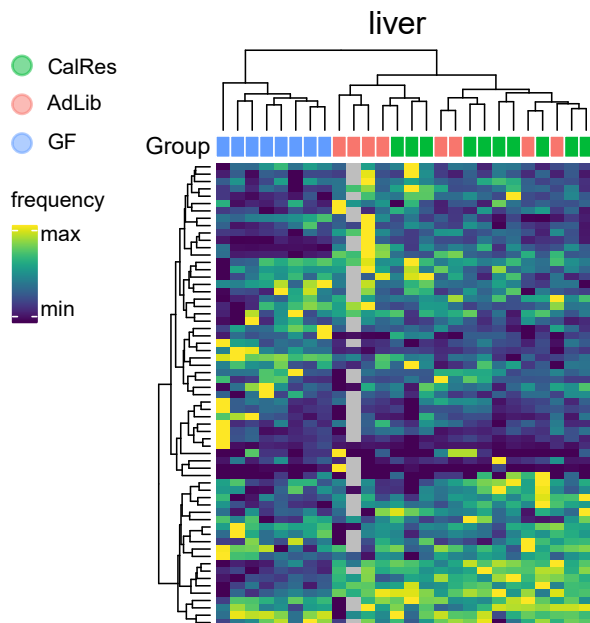**B**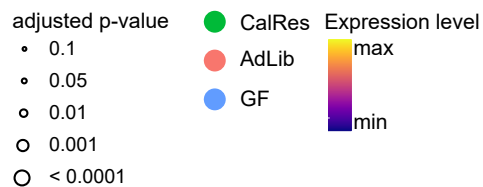**C**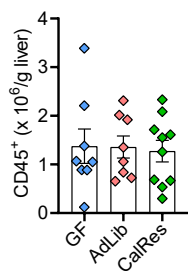**D**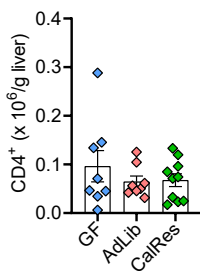**E**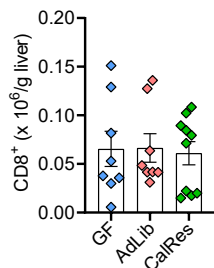**F**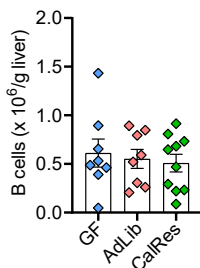**G**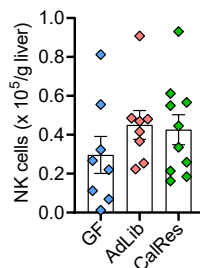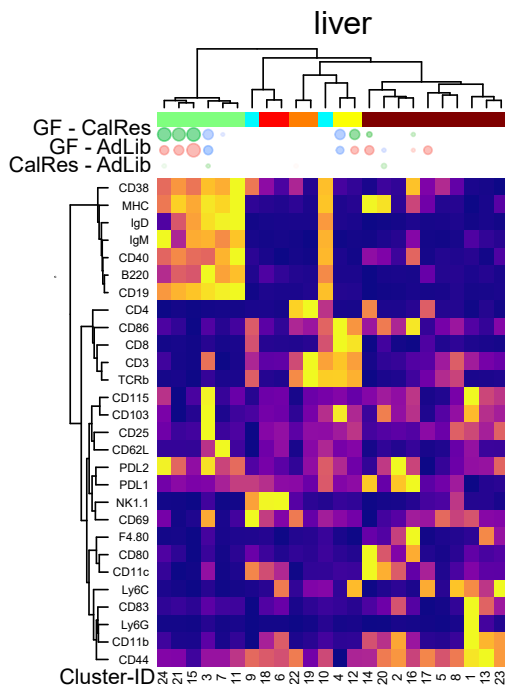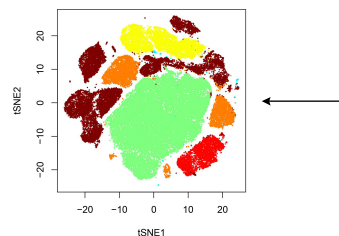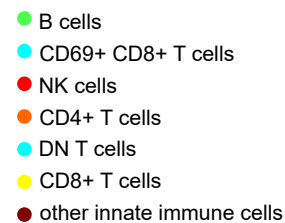

Supplement: Supplementary file 2 — Additional file 1: Supplementary Figure 1. Related to Fig. 1. MMSP50 is a representative donor of the weight loss cohort. A Examination of baseline alpha diversity demonstrates that MMSP50 is at the 54th ranked percentile for baseline diversity after VLCD. B Their baseline microbiota composition (principal coordinates analysis of Bray-Curtis Dissimilarity) is well within the 95% confidence interval of baseline composition for the cohort (dotted line) and C their change in community structure is the 19th percentile for change in composition. Supplementary Figure 2. Related to Fig. 2. No significant changes in energy loss or fecal content after microbial colonization. Metabolic analysis of germ-free (GF) mice and mice inoculated with the AdLib and CalRes human gut microbiota. A-D Energy loss (A), fecal energy content (B), food consumption (C), and energy absorption (D) were measured using bomb calorimetry in GF and colonized mice. E Body weights in g. ** P < 0.01, *** P < 0.001 as determined using 2-way ANOVA with Bonferonni’s post-test correction for multiple comparisons. error bars = SEM. Supplementary Figure 3. Related to Fig. 3. Differential expression of surface markers in different colonic immune cell clusters of germ-free and colonized mice. A The heatmap shows differentially distributed colonic immune cell phenotypes quantified by PhenoGraph clustering. The distribution of each cell cluster (rows) is shown for each murine sample (columns). B The heatmap shows the distribution of colonic immune lineages based on the expression of canonical lineage markers by t-SNE on all colonic viable CD45+ leukocytes. The differential expression of each selected surface marker (rows) is shown for each immune cell cluster (columns). The significance levels of the comparison between the groups for each immune cell cluster are depicted by semi-supervised hierarchical clustering. The top bubbles denote clusters with significantly different abundances between the groups. Bubble co [file 40168_2022_1249_MOESM2_ESM.zip › Supplementary Figure 5.pdf]

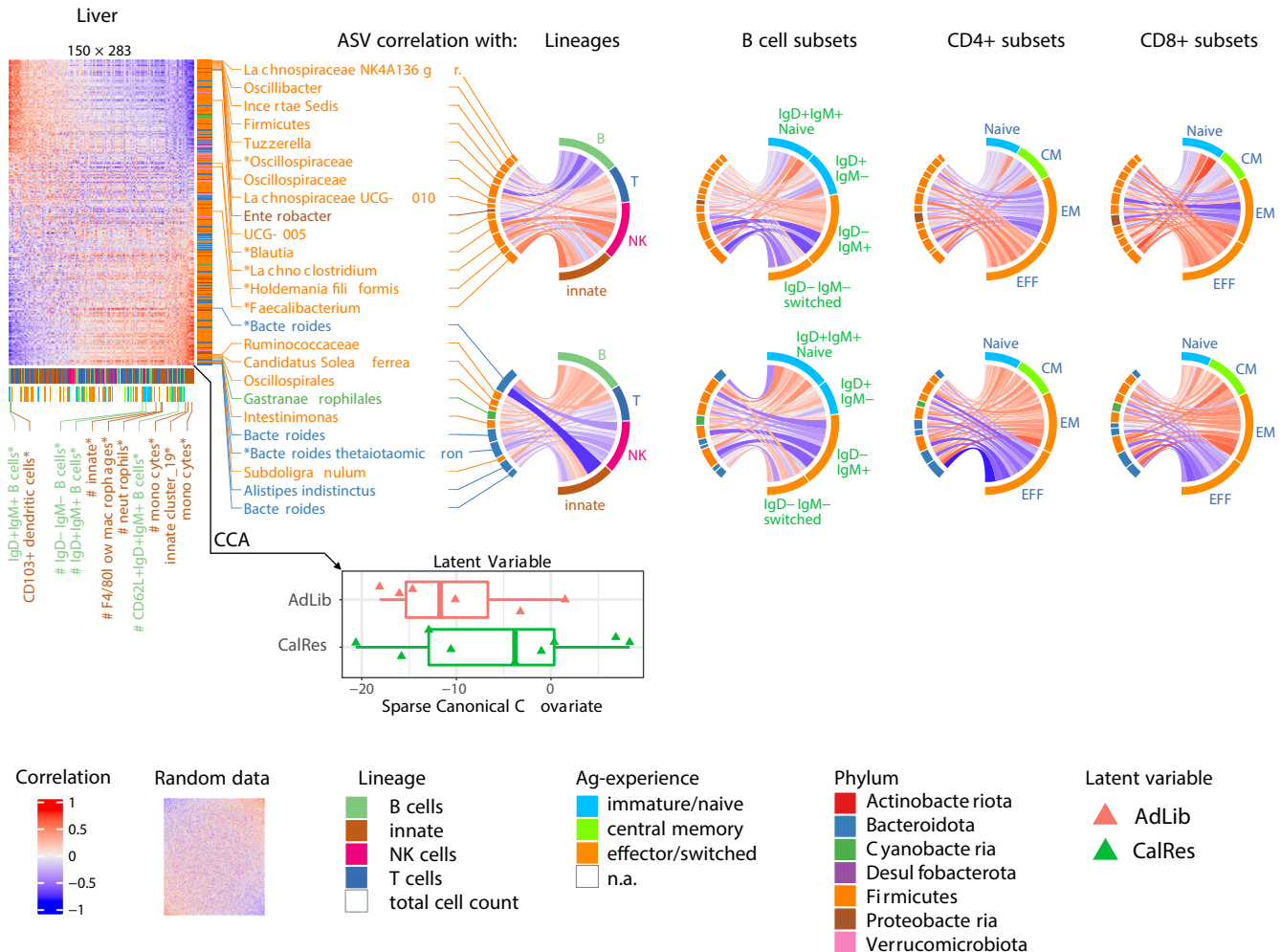

Supplement: Supplementary file 2 — Additional file 1: Supplementary Figure 1. Related to Fig. 1. MMSP50 is a representative donor of the weight loss cohort. A Examination of baseline alpha diversity demonstrates that MMSP50 is at the 54th ranked percentile for baseline diversity after VLCD. B Their baseline microbiota composition (principal coordinates analysis of Bray-Curtis Dissimilarity) is well within the 95% confidence interval of baseline composition for the cohort (dotted line) and C their change in community structure is the 19th percentile for change in composition. Supplementary Figure 2. Related to Fig. 2. No significant changes in energy loss or fecal content after microbial colonization. Metabolic analysis of germ-free (GF) mice and mice inoculated with the AdLib and CalRes human gut microbiota. A-D Energy loss (A), fecal energy content (B), food consumption (C), and energy absorption (D) were measured using bomb calorimetry in GF and colonized mice. E Body weights in g. ** P < 0.01, *** P < 0.001 as determined using 2-way ANOVA with Bonferonni’s post-test correction for multiple comparisons. error bars = SEM. Supplementary Figure 3. Related to Fig. 3. Differential expression of surface markers in different colonic immune cell clusters of germ-free and colonized mice. A The heatmap shows differentially distributed colonic immune cell phenotypes quantified by PhenoGraph clustering. The distribution of each cell cluster (rows) is shown for each murine sample (columns). B The heatmap shows the distribution of colonic immune lineages based on the expression of canonical lineage markers by t-SNE on all colonic viable CD45+ leukocytes. The differential expression of each selected surface marker (rows) is shown for each immune cell cluster (columns). The significance levels of the comparison between the groups for each immune cell cluster are depicted by semi-supervised hierarchical clustering. The top bubbles denote clusters with significantly different abundances between the groups. Bubble co [file 40168_2022_1249_MOESM2_ESM.zip › Supplementary Figure 6.pdf]
